# Early Initiation of Breastfeeding Among Adolescent Mothers: A Quality Improvement Study

**DOI:** 10.7759/cureus.31716

**Published:** 2022-11-20

**Authors:** Avir Sarkar, Sivaranjani P Selvam, Anjaly Raj, Isha Wadhawan, Ramesh Chandra

**Affiliations:** 1 Obstetrics and Gynecology, Employee's State Insurance Corporation (ESIC) Medical College and Hospital, Faridabad, IND; 2 Obstetrics and Gynecology, All India Institute of Medical Sciences, Kalyani, IND

**Keywords:** teenage pregnancy, breastfeeding, quality improvement, early initiation of breastfeeding, adolescent pregnancy

## Abstract

Background: The prevalence of early initiation of breastfeeding (EIBF) was found to be low among adolescent postnatal mothers. EIBF is associated with improved parenting skills and better neurodevelopment in babies. We aimed to improve the prevalence of EIBF among teenage mothers to at least 90% through a quality improvement (QI) initiative.

Materials and methods: It was a QI intervention conducted over a period of 15 months involving mothers under the age of 20 years at the time of present delivery. Six Plan-Do-Study-Act (PDSA) cycles consisting of multiple interventions, each lasting for one month, were conducted to improve the prevalence of EIBF. Rate of improvement in EIBF was noted and plotted against time. Post-intervention follow-ups of observations were done for six months.

Results: The prevalence of EIBF among adolescent mothers was 28.5% during the pre-intervention baseline phase. QI team meeting was held and the barriers to EIBF among the adolescent mothers were discussed and depicted in the form of a fish-bone analysis model. The prevalence of EIBF increased during each intervention cycle to 50%, 60%, 62.5%, 72.7%, 88%, and 100%. At the end of six months follow-up phase, the prevalence of EIBF sustained at around 100%.

Conclusion: This QI initiative has proven to be effective in improving the prevalence of EIBF with simple but effective measures. Adolescent women comprise of a vulnerable sub-population of high-risk mothers. Proper counseling and respectful maternity care will help them face the challenges of motherhood boldly.

## Introduction

Early initiation of breastfeeding (EIBF) is known to reduce neonatal mortality and morbidity [1,2]. Colostrum, being a rich source of lymphocytes and immunoglobulins, helps in providing a boost to the developing immune system of the newborn [3]. EIBF maintains normothermia, prevents neonatal diarrhea, and accelerates uterine involution, thereby reducing chances of postpartum hemorrhage [4]. Despite the fact that about one million neonates can be saved from death by EIBF in the first hour of life, global estimates state that the rate of EIBF ranges from 17.7% to 57.6% across various countries [2,5]. As countries around the globe have now achieved good targets of institutional deliveries, more and more focus needs to be exerted on EIBF and skin-to-skin contact in the first hour after delivery [6]. Adolescent pregnancies are generally associated with a plethora of antenatal and postpartum complications, among which, difficulty in initiation of breastfeeding is a common problem. This quality improvement (QI) initiative was undertaken to gear up the prevalence of EIBF among adolescent mothers using standard continuous QI methods of root cause analysis and Plan-Do-Study-Act (PDSA) cycles.

## Materials and methods

This QI initiative was conducted at a tertiary care teaching hospital in North India over a period of 15 months. It aimed to improve EIBF among neonates born to adolescent mothers. A baseline cross-sectional assessment over a period of three months showed that the rate of EIBF (within one hour of delivery) was low among teenage mothers compared to the adult population. Institutional ethics committee approval was obtained from Employee's State Insurance Corporation (ESIC) Medical College and Hospital, Faridabad, prior to the commencement of the study (approval number: 134/X/11/13/2021-IEC/36). Written informed consent was obtained from all participants. The study was conducted in the following three phases: a pre-intervention baseline phase (three months), an intervention phase (six months), and a post-intervention follow-up phase (six months).

**Figure 1:**
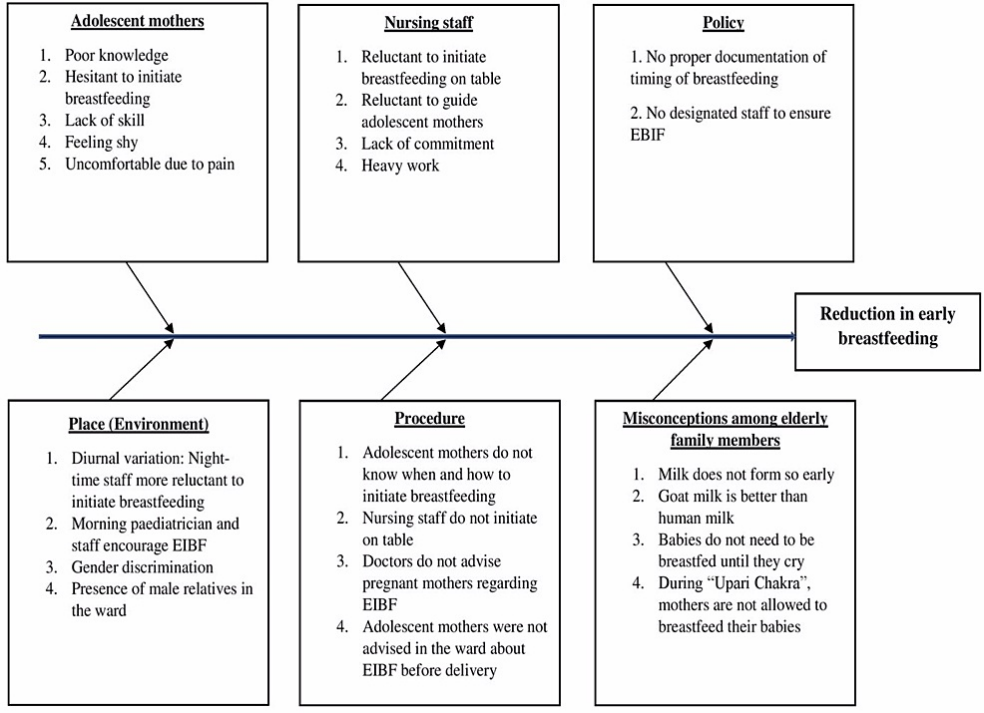
Fish-bone analysis of the barriers to early initiation of breastfeeding among teenage mothers. EIBF: early initiation of breastfeeding

All deliveries being conducted (both through vaginal and abdominal routes) during the study time frame, where the newborns were shifted to mothers' side after initial resuscitation by the concerned pediatrician, were potentially eligible for recruitment. Mothers under the age of 20 years at the time of present delivery were included in the study. For women below 18 years, written assent was obtained. The cases where newborns required intensive care unit admission in the first hour of life or referral to other higher centers and babies born to mothers of age 20 years or more at the time of delivery were excluded from the study. During the pre-intervention phase, a baseline audit was conducted over a period of three months to assess the prevalence of EIBF among adolescent mothers delivering at our hospital. A QI team was constituted of six members under the guidance of the team leader. Team members included faculty guides, resident doctors, and nursing staff in the labor and postnatal wards. In the first meeting, the dedicated QI team formulated the problem statement, smart aim, and strategies to implement interventions. The problem statement tried to address the low rate of EIBF among teenage mothers in our hospital setup. The issues were poor knowledge, hesitation to initiate breastfeeding, lack of skill, and shyness at the level of adolescent mothers, and reluctance to initiate and guide the adolescent mothers at the level of nursing staff. No proper documentation of timing of breastfeeding and no designated staff to ensure EIBF were hospital-level policy issues. Presence of male relatives in the ward and gender discrimination were also noted. The smart aim was to increase the prevalence of EIBF to at least 90% through dedicated PDSA cycle. Each PDSA cycle included these various methods - (a) monthly training of the doctors and nursing staff was conducted through dedicated lectures and interactive question and answer sessions, (b) documentation of EIBF in the safe birth checklist along with mention of the interval between delivery and initiation of breastfeeding, (c) building a separate cubicle for postpartum mothers for breastfeeding counseling, (d) display of posters and charts depicting the importance and advantages of EIBF, and (e) counseling sessions for the antenatal women and their birth companions in the labor room when they got admitted with spontaneous onset of pain or for induction of labor. After the collection of baseline observations, a second team meeting was conducted and the possible reasons for the low prevalence of EIBF were discussed in detail. A conceptual framework of the possible reasons for low EIBF was noted and the same was plotted in the form of a fish-bone analysis chart (Figure 1). Team leader finalized the strategies for conducting the PDSA cycle to improve the low rate of EIBF in the first hour after delivery among teenage mothers.

At the beginning of the intervention phase, the team members identified their respective roles. The nursing staff played a central role in implementing EIBF among the recruited population. Resident doctors and consultants in charge of the labor wards were given the role of training and supporting the nursing staff with antenatal and postpartum counseling and universal implementation of EIBF after delivery. The nursing in charge of the labor room and postpartum wards would supervise all the staff on duty and ensure strict adherence to the proposed protocols. For bringing out the changes, various methods were employed in the PDSA cycles - (a) monthly training of the doctors and nursing staff was conducted through dedicated lectures, interactive question, and answer sessions; (b) documentation of EIBF in the safe birth checklist along with mention of the interval between delivery and initiation of breastfeeding; (c) building a separate cubicle for postpartum mothers for breastfeeding counseling; (d) display of posters and charts depicting the importance and advantages of EIBF; and (e) counseling sessions for the antenatal women and their birth companions in the labor room when they got admitted with spontaneous onset of pain or for induction of labor.

Outcome measures were assessed by recording the monthly percentages of newborns breastfeeding within one hour of delivery (either by vaginal or abdominal route), to assess the effect of interventions. Outcomes were noted over a period of six months. During this time, monthly training of the doctors and nursing staff was being undertaken regularly (on the first of each month). Data collectors (all six team members) maintained records of the total number of deliveries conducted in adolescent women and the number of cases where EIBF was initiated. In the post-intervention follow-up phase, which lasted for six months, only observations were done without any intervention. All data were entered in a Microsoft Excel matrix and analyzed using IBM Statistical Package for Social Sciences Statistics, version 20.0.0 (Armonk, NY: IBM Corp.). Categorical variables were expressed in percentages and frequencies. The monthly rate of EIBF expressed in percentages was plotted in a line chart for better representation and comparison over the period of time.

## Results

**Figure 2:**
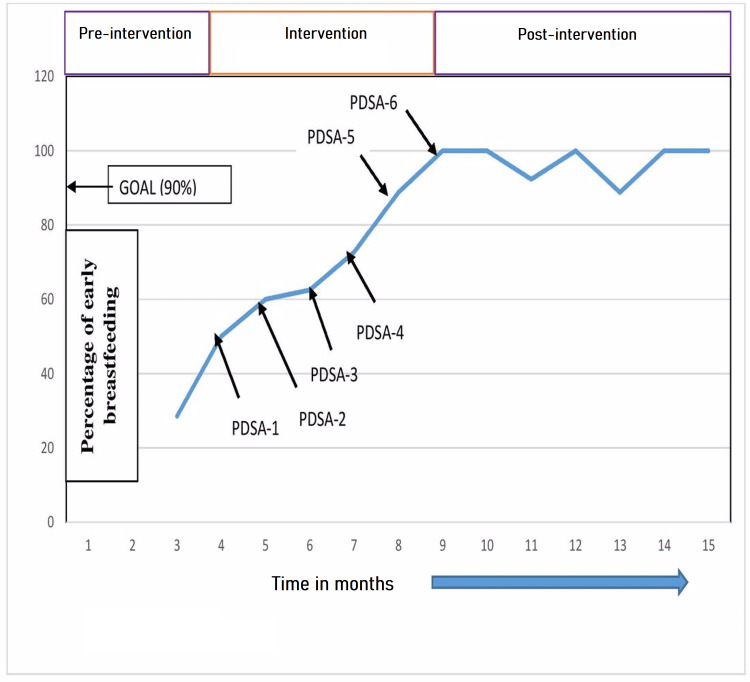
Line chart showing the increase in early breastfeeding after delivery among teenage mothers during the study time frame. PDSA: Plan-Do-Study-Act

Overall 122 adolescent deliveries were included in the study, starting from the pre-intervention phase to the post-intervention phase. The prevalence of EIBF over the three months of pre-intervention baseline phase was only 28.5% (Figure 2). At the end of pre-intervention phase, the reasons for the low prevalence of EIBF were analyzed at all levels starting from the postpartum mothers to the nursing staff, hospital environment, social misconceptions, and hospital protocols. The barriers were depicted through a fish-bone model as shown in Figure 1. The intervention phase included six PDSA cycles, each lasting for one month. There was a progressive increase in the prevalence of EIBF after each PDSA cycle (Figure 2). The prevalence of EIBF increased to 50%, 60%, 62.5%, 72.7%, 88.8%, and 100% after each successive intervention cycle (Figure 2). During the post-intervention follow-up phase of six months, the prevalence dropped to 88.8% (at the end of fourth month) but again increased to 100% by the end of sixth month. So good sustenance of more than 90% EIBF could be achieved through this QI intervention.

## Discussion

This QI initiative aimed to improve the EIBF among adolescent mothers at a tertiary care hospital using the standard techniques of root cause analysis and PDSA cycles. A QI initiative conducted to improve early breastfeeding among newborns delivered in a secondary care hospital in Northern India increased the prevalence of EIBF from 52% to 97% [7]. Another QI initiative to improve the rate of first-hour breastfeeding of newborns also proved effective by increasing its prevalence from 12% to 80% [8]. The prevalence of first-hour breastfeeding among postnatal mothers in another study conducted in a tertiary care hospital in India was 21.5% [9]. Similarly, the prevalence of EIBF among adolescent mothers of our study during the baseline phase was 28.5% only. Particularly among adolescent mothers, there existed a lack of knowledge regarding when and how to breastfeed their newborns. Literature reports that adolescent mothers face great difficulty in timely initiating breastfeeding and this is one of the important reasons for a huge number of neonatal intensive care unit admission of babies born to these women [10]. Adolescent mothers need extra efforts from the nursing staff in terms of counseling, support, and encouragement. There exists a reluctance from the nursing staff and resident doctors regarding counseling and training adolescent mothers which require some more time compared to adult postnatal mothers. Lack of commitment and heavy workload in a tertiary care hospital has been proposed to be the reasons for low prevalence of EIBF. So the intervention phase started with regressive training of resident doctors and nursing staff posted in the labor and postnatal wards. According to National Family Health Survey, the prevalence of EIBF in India is 41.6% [11]. Worldwide, around one million newborns could be saved with first-hour initiation of breastfeeding [11]. EIBF has many maternal and neonatal advantages. As the mother holds and feeds her baby it improves the parental skills and empowers the mother. It is also associated with better neural development of the baby. Postnatal mothers need proper guidance, support, and encouragement to achieve EIBF as suggested by baby-friendly hospital initiative [12]. We advocated these findings with display of posters and charts depicting the advantages of breastfeeding to kindle and nurture the practice of EIBF.

Like other QI initiatives which showed positive outcomes, our study also proved effective in improving EIBF despite the presence of so many hurdles. There were no proper hospital policies for EIBF, no documentation of timing of breastfeeding post-delivery, and no designated personnel to insist and supervise EIBF. With our QI initiative, nursing staff and resident doctors were trained and encouraged to insist on EIBF under the supervision of the nursing in charge of the postnatal wards. Dudeja et al. employed the same model of QI initiative which improved the first-hour breastfeeding after cesarean delivery from zero to 93% over a period of three months [13]. The PDSA cycles aided us in executing and assessing the initiatives gradually.

The strength of this study was that it included consultants, resident doctors, and nursing staff as a designated team undergoing rigorous training every month. All were involved in learning and teaching through active participation. It involved simple but most effective measures to improve the EIBF which can be employed in all healthcare centers with low-resource settings. The major limitation was it was single-centered. Owing to the huge patient load, maternal satisfaction could not be assessed in this study. Moreover, counseling of the family members and birth attendants could not be done effectively during the study period.

## Conclusions

This QI initiative was very effective in improving EIBF among adolescent mothers, thereby reducing neonatal morbidity and mortality. A designated team involving obstetric consultants, resident doctors, and nursing staff could improve breastfeeding initiation and overall maternal behavior among teenage mothers. The employment of simple steps by displaying posters and charts depicting the advantages of EIBF has proven effective in our hospital setting. It can be employed in low-resource health settings where teenage pregnancies are still a burden to sustainable development of women.
